# Species composition, richness, and distribution of marine bivalve molluscs in Bahía de Mazatlán, México

**DOI:** 10.3897/zookeys.399.6256

**Published:** 2014-04-08

**Authors:** María del Carmen Esqueda-González, Eduardo Ríos-Jara, Cristian Moises Galván-Villa, Fabian Alejandro Rodríguez-Zaragoza

**Affiliations:** 1 Laboratorio de Ecosistemas Marinos y Acuicultura, Departamento de Ecología, Centro Universitario de Ciencias Biológicas y Agropecuarias, Universidad de Guadalajara. Las Agujas Nextipac, Zapopan, Jalisco, México. C.P.45110

**Keywords:** Mollusca, Bivalves, Taxonomic distinctness, Bahía de Mazatlán, Mexican Pacific

## Abstract

We describe the composition and distribution of bivalve molluscs from the sandy and rocky intertidal and the shallow subtidal environments of Bahía de Mazatlán, México. The bivalve fauna of the bay is represented by 89 living species in 28 families, including 37 new records and four range extensions: *Lithophaga hastasia*, *Adula soleniformis*, *Mactrellona subalata*, and *Strigilla ervilia*. The number of species increases from the upper (44) and lower intertidal (53) to the shallow subtidal (76), but only 11 (17%) have a wide distribution in the bay (i.e., found in all sampling sites and environments). The bivalve assemblages are composed of four main life forms: 27 epifaunal species, 26 infaunal, 16 semi-infaunal, and 20 endolithic. A taxonomic distinctness analysis identified the sampling sites and environments that contribute the most to the taxonomic diversity (species to suborder categories) of the bay. The present work increased significantly (31%) to 132 species previous inventories of bivalves of Bahía de Mazatlán. These species represent 34% of the bivalve diversity of the southern Golfo de California and approximately 15% of the Eastern Tropical Pacific region.

## Introduction

Studies on molluscs from Bahía de Mazatlán, located in the Mexican Pacific, have focused mainly on the conspicuous species of gastropods and bivalves from the rocky intertidal (Arreguín-Romero 1982, Sánchez-Vargas 1984, Olabarria et al. 2001, Camacho-Montoya et al. 2007, Vega et al. 2008, Rendón-Díaz 2010) and rocky-sandy subtidal environments (Parker 1963, Orozco-Romo 1980, Sánchez-Vargas 1984). Altogether these studies have reported 83 species of bivalves. However there has never been a complete inventory, as there are many inconspicuous infaunal, semi-infaunal, and endolithic forms which have been recorded elsewhere in the Mexican Pacific region (Keen 1971, Keen and Coan 1974, Hendrickx and Toledano-Granados 1994, Hendrickx and Brusca 2002, Ríos-Jara et al. 2008), but not collected yet in Bahía de Mazatlán. For example, Parker (1963) recorded 380 bivalve species only in the Golfo de California; Coan (1968) recorded 75 species in Bahía de los Ángeles, located in the northern portion of this gulf; Hendrickx et al. (2007) recorded up to 565 species; Keen (1971) listed 567 species for the Panamic Province.

According to Bouchet et al. (2002), mollusc species richness has been frequently underestimated in ecological studies mainly because of an inadequate coverage of the spatial heterogeneity and sampling effort. This is particularly important in the case of bivalves because they possess a wide variety of life forms and exploit a large number of habitats, which require specialized sampling techniques. Thus, special consideration should be given to the complexity of the environment and to sampling techniques in order to obtain a better understanding of the structure of the assemblages.

Many studies on marine biodiversity have used species accumulation curves (i.e., sample-based rarefactions) to evaluate the sampling effort; this technique indicates when a sufficiently large percentage of species has been observed with a definite number of samples with respect to a theoretical expected total number of species of a given community (Magurran 2004). The evaluation of sampling effort is particularly important in the case of molluscan assemblages, which often contain a large number of rare species, including unique (recorded in only one sample) (Bouchet et al. 2002) and duplicate species (recorded only in two samples). Therefore, the use of different estimators has been recommended when many unique and duplicate species are found in a large set of samples since they have complementary characteristics (Escalante-Espinoza 2003, Magurran 2004).

Marine biodiversity has been evaluated with the taxonomic distinctness approach (Warwick and Light 2002, Clarke and Gorley 2006), which integrates the species richness and all taxonomic categories of an assemblage of species. The average taxonomic distinctness measures the extent to which the species in a sample are taxonomically related. This is the average taxonomic distance between all pairs of species across a taxonomic tree. This analysis determines the extent to which certain taxa contribute to the total diversity of a certain environment or site using only the species presence-absence data, and it is insensitive to differences in sampling effort and sampling techniques used across different scales (Clarke and Warwick 1999). The assessment of biodiversity at a regional scale is occasionally difficult, but the taxonomic distinctness parameter facilitates this measurement (Warwick and Light 2002). Furthermore, the average taxonomic distinctness indexes (Δ^+^ and Λ^+^) are an easy-to-use tool to measure biodiversity in the time and space scales (Warwick and Clarke 1998), as confirmed in studies on fish communities (Roger et al. 1999), macrobenthic communities (Mistri et al. 2000), marine nematodes (Clarke and Warwick 2001), assemblages of empty molluscan shells (Warwick and Light 2002, Smith 2008), freshwater organisms (Heino et al. 2005), and aquatic insects (Heino et al. 2008).

Bahía de Mazatlán is located in the southern portion of Golfo de California. The alternating warm and temperate seasons of this region create conditions that favor the development of a very diverse marine biota composed by species from both Golfo de California and the Mexican Tropical Pacific biogeographic subprovinces (Brown and Lomolino 1998). In this work we describe the taxonomic composition of the bivalve communities inhabiting the intertidal and shallow subtidal (depths 3–10 m) environments from four rocky and two sandy shores of Bahía de Mazatlán. Bivalve specimens were collected using various sampling techniques and during different seasons of the year to obtain a good representation of the epifaunal, infaunal, semi-infaunal, and endolithic species of the bay. Species accumulation curves were used to evaluate the sampling effort performed during the study period, and to predict the theoretical expected total number of bivalve species of these environments. We also provide a comparative analysis of previous inventories performed in the bay, the new records and the geographical range extensions. Finally, the taxonomic composition is analyzed using the average taxonomic distinctness index and its variation to evaluate the variability in the composition and distribution of the different taxonomic categories from the species to the subordinal level.

## Material and methods

**Figure 1.**
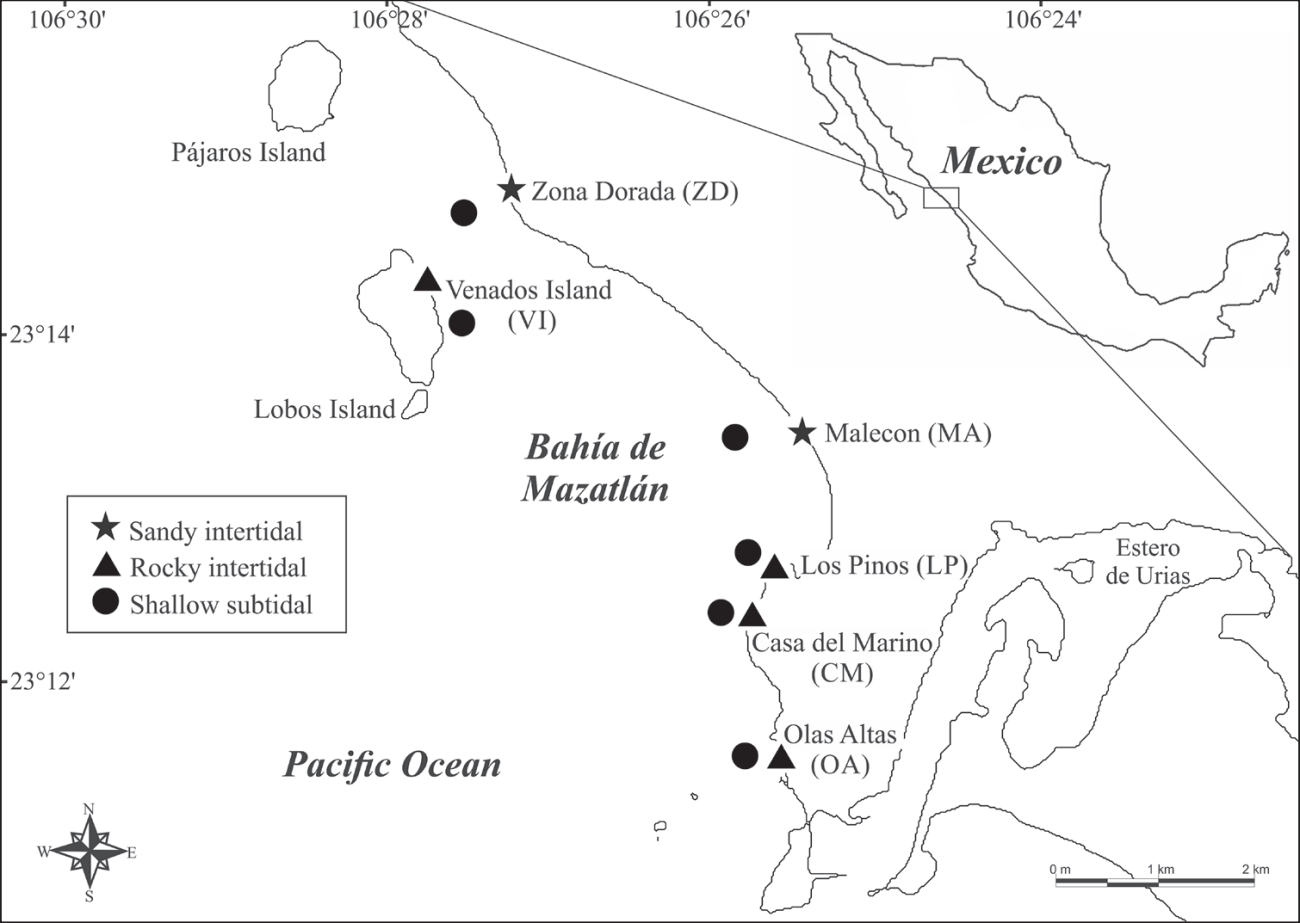
Study area and sampling sites at Bahía de Mazatlán, México.

*Study Area*. Bahía de Mazatlán is located at the mouth of the Golfo de California (23°15'–23°11'N, 106°29'–106°25'W) (Figure 1). The bay has a total extent of approx. 3,500 hectares and a coastline of 13.5 km. There are three major islands (Venados, Pájaros, and Lobos), located approximately 1.5 km off the coast. These islands are protected as ecological reserves for migratory birds and marine animals and plants, and part of the “Islands of the Golfo de California Protection Area” (CONABIO 2012).

The bay belongs to the Cortesian Eco-Region included in the Warm-Temperate Northeast Pacific Province (Spalding et al. 2007). It is seasonally influenced by the California Current with cold water from the north, the North-Equatorial Countercurrent with flow of warm tropical waters, and the temperate waters from Golfo de California itself (Wyrtki 1966, Zamudio et al. 2001, Alonso-Rodríguez 2004). The climate is tropical-subtropical with two very distinct seasons (Bell and Carballo 2008). The wet season occurs from July to October, and the dry season, with little or no rainfall, occurs from November to June (Montaño-Ley 1985). The sea surface temperature ranges from 13–21 °C in the dry season and from 28–31 °C in the wet season (Wilkinson et al. 2009).

*Fieldwork*. Six sampling sites protected or exposed to wave action conditions were established along Bahía de Mazatlán: four rocky beaches and two sandy beaches. Three environments were considered in each site: upper intertidal (UI), lower intertidal (LI), and shallow subtidal (SS) (3–10 m depth) adjacent to each beach. The intertidal zones were defined according to the natural zonation of benthic invertebrates (Peres 1982, Sánchez-Vargas 1984, Esqueda et al. 2000).

Some of the general characteristics of these beaches are: 1) **Olas Altas** (OA), a partially-protected beach with a well-developed rocky area of approx. 150 m long and smaller areas of medium-to-coarse sand; 2) **Los Pinos** (LP), a protected rocky beach approx. 100 m long, with some areas of medium-to-coarse sand; 3) **Casa del Marino** (CM), a semi-protected to exposed rocky beach approx. 250 m long mixed with small sandy areas of fine-to-medium size grains; 4) **Venados Island** (VI), the east side of the island has an extensive protected sandy beach approx. 850 m long and 30–60 m wide with medium-to-coarse sand; towards the northern part of the beach there is a rocky beach approx. 200 m long with many tidal pools and boulders; 5) **Malecon** (MA), an exposed and very dynamic sandy beach approx. 400 m long of medium-to-coarse sand and pebbles. The beach runs along the urban sector of the city of Mazatlán and it is frequently visited by locals and tourists; and 6) **Zona Dorada** (ZD), a protected beach of medium-to-fine sand approx. 300 m long located in the tourist hub of the city just in front of hotels and extensively visited by tourists. The adjacent shallow subtidal environments of all these beaches include mixed substrates composed by coarse and fine sand, rocky reef areas, and many shell fragments. In Venados Island and Los Pinos small patches of live coral are also frequent.

Different sampling techniques (transect-quadrats, dredges, and direct searches) were applied during four expeditions in December 2008, and March, June, and August 2009. The transects (15 m long) were set parallel to the coastline, two in each environment (UI, LI, SS) of each beach. Two to four (x = 3) quadrats (0.5 m^2^) were placed equidistant along each transect and all bivalves found in each quadrat identified in situ or collected for taxonomic identification in the laboratory. In the shallow subtidal, sampling was performed during SCUBA diving. The total sampling effort was 126 quadrats (63 m^2^) in the rocky intertidal and 52 quadrats (26 m^2^) in the sandy intertidal. A total of 90 quadrats (45 m^2^) were sampled in the shallow subtidal environments of all beaches. Additionally, in order to increase the inventory of bivalves, the specimens found in the areas immediately adjacent to the quadrats were also identified *in situ* or collected during direct searches in the intertidal and shallow subtidal environments. Dredges (24) were carried out in the shallow subtidal zone (8–15 m depth) of the six beaches, using a naturalist´s dredge (mesh size = 2.5 cm, cod-end mesh size = 1.3 cm) (English et al. 1997) during 15 min. at an approximate speed of 2 knots.

*Laboratory methods*. A detailed examination of each sample was conducted to search for bivalves. Only living specimens were considered. Endolithic specimens (i.e., those growing within rocks or other hard substrates) were obtained by breaking rocks and shells, coral fragments, polychaete tubes, and rodoliths. Epifaunal specimens (i.e., species attached to a hard substrate) were obtained by scraping the surface of rocks. Semi-infaunal specimens (i.e., partially buried in the sediment but protruding above it) and infaunal specimens (< 4 mm) (i.e., those living buried in soft substrate) were obtained by screening the sandy sediment (Levinton 2001). A stereo microscope was used for examining soft and hard substrates in search of specimens < 10 mm and for taxonomic determination. The following references were used for the taxonomic identification of bivalves: Abbott (1974), Keen (1971), Morris (1980), and Coan and Valentich-Scott (2012). Previous inventories, additions, and taxonomic changes which include records from Bahía de Mazatlán were also reviewed (Parker 1963, Keen 1971, Orozco-Romo 1980, Arreguín-Romero 1982, Sánchez-Vargas 1984, Skoglund 2001, Olabarria et al. 2001, Hendrickx et al. 2005, Camacho-Montoya et al. 2007, Rendón-Díaz 2010, Coan and Valentich-Scott 2012).

The absolute frequency of every species in each environment and site was estimated by calculating the ratio between the number of sites where that species was recorded and the total number of sites. A reference collection was set up with all the locality information in the Laboratory of Marine Ecosystems and Aquaculture at the Department of Ecology, University of Guadalajara, México. Voucher specimens were also deposited in this laboratory.

*Analysis of the data*. Only specimens recorded with the transect-quadrat method in the rocky intertidal and the adjacent shallow subtidal zones were used to evaluate sampling effort. Species accumulation curves were based on the cumulative number of species per quadrat. The expected richness was calculated using the nonparametric estimators Chao 2, Jackknife 1, and Jackknife 2. Plots were constructed with 10,000 non-replacement iterations based on samples for each site and environment, using the software EstimateS v8 (Colwell 2006).

A species presence-absence matrix was constructed using information from the records obtained from the transect-quadrats, dredges, and direct search techniques on the rocky beaches (Olas Altas, Los Pinos, Casa del Marino, and Venados Island). Six taxonomic levels (species, genus, family, superfamily, order, and superorder) were considered based on the classification schemes of Coan and Valentich-Scott (2012) (species to family) and Bouchet and Rocroi (2010) (superfamily to superorder). These taxa were weighted according to Warwick and Clarke (1995), as follows: w1, species within the same genus; w2, species within the same family but in different genera; w3, species within the same superfamily but in different family; w4, species within the same order but in a different superfamily; and so on. The average taxonomic distinctness Δ^+^ and its variation Λ^+^ were estimated for each site, environment and site-by-environment combination. Models were made with 95% confidence intervals, and the statistical significance of Δ^+^ and Λ^+^ were tested using 1,000 permutations in the program PRIMER v6 + PERMANOVA (Clarke and Gorley 2006).

## Results

### Species accumulation curves

**Figure 2.**
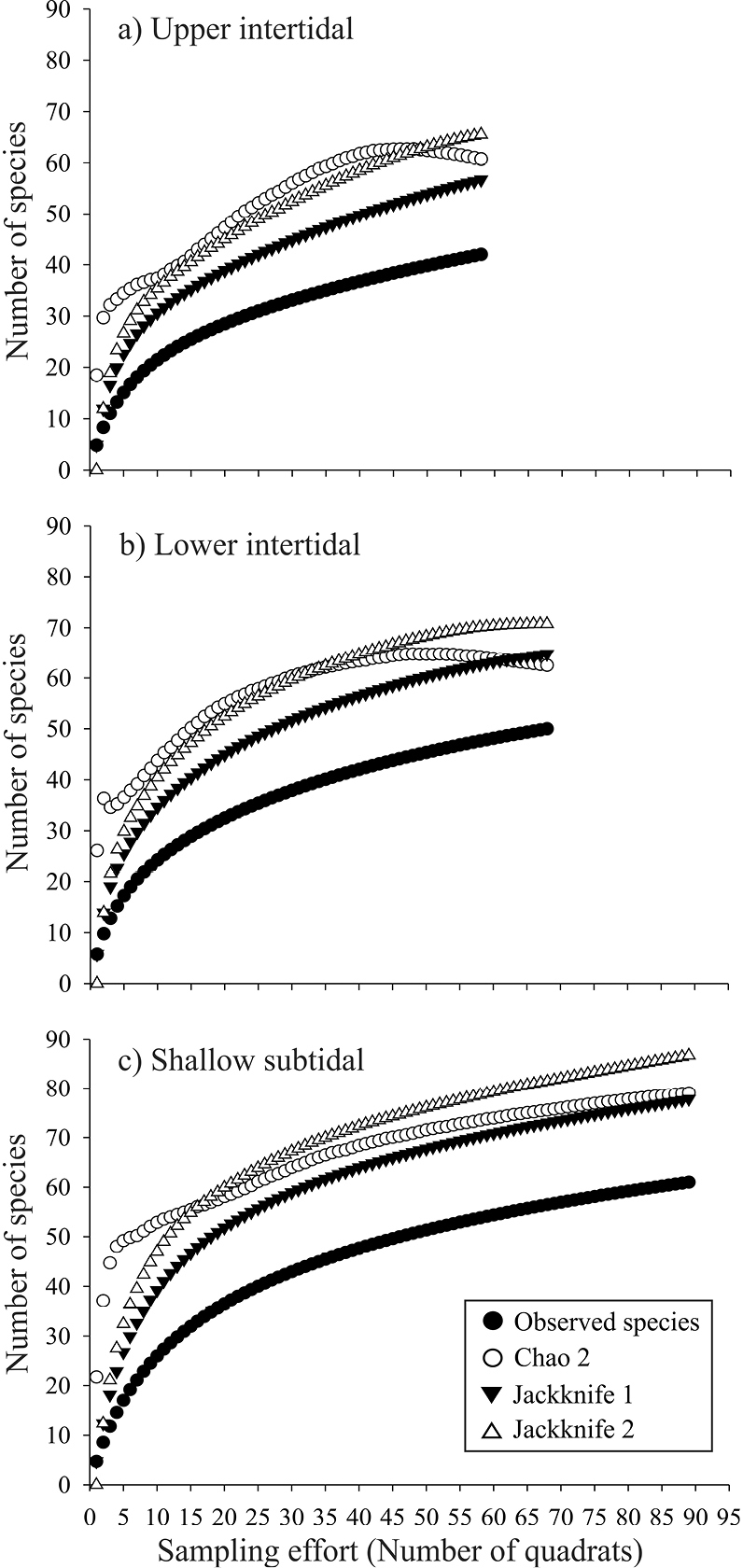
Observed and expected bivalves species accumulation curves, with nonparametric indices Chao 2, Jackknife 1, and Jackknife 2, in the three environments of Bahía de Mazatlán (**a–c**). Plots were constructed with 10,000 non-replacement iterations.

**Figure 3.**
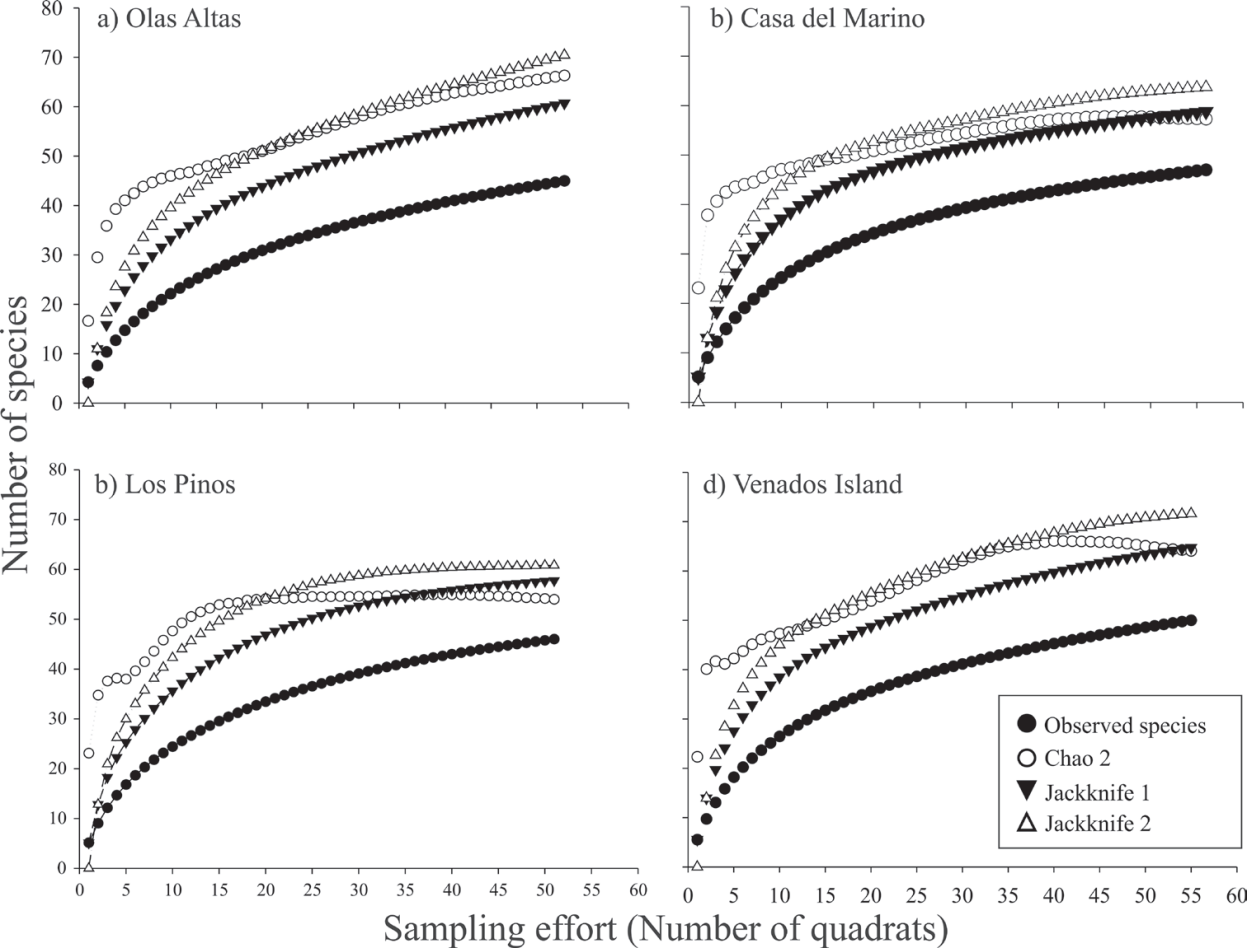
Observed and expected bivalves species accumulation curves, with nonparametric indices Chao 2, Jackknife 1, and Jackknife 2, of four sites of Bahía de Mazatlán (**a–d**). Plots were constructed with 10,000 non-replacement iterations.

The species accumulation curves show a trend towards an symptote in all environments (Figure 2). The observed species representativeness with respect to the estimators Chao 2, Jackknife 1, and Jackknife 2 ranged between 64 and 80%, with Jackknife 1 always estimating the lowest expected richness, while Jackknife 2 always estimating the highest values. The species accumulation curves revealed a similar and more evident trend in the four sampling sites (Figure 3). The observed species representativeness ranged between 64 and 85% with respect to the estimators, with Jackknife 2 being the estimator that yielded the highest expected richness. Los Pinos and Casa del Marino possessed the highest species representativeness (≥ 79%), while Olas Altas had the lowest value (68-75%). A large number of unique (12–17) and duplicate (6–9) species were obtained in the three environments and the four sites (Table 1).

**Table 1.** Rarity of species in four sites and three environments in Bahía de Mazatlán, México.

Rarity of species	Sites				Environments		
	Olas Altas	Los Pinos	Casa Marino	Venados Island	Upper intertidal	Lower intertidal	Shallow subtidal
Uniques	16	12	12	15	15	15	17
Duplicates	6	9	7	8	6	9	8

### Richness, composition, and distribution of species

A total of 21,694 live bivalve specimens was recorded, representing 28 families, 55 genera, and 89 species (Table 2). The most diverse families were Mytilidae (14 species), Veneridae (10), and Arcidae (8). Ten families (35%) included only one species. The number of species increased from the upper (44) and lower intertidal (53) to the shallow subtidal (76). In addition, the numbers of unique species were 7 (upper), 4 (lower), and 18 (subtidal). The species richness was similar in the adjacent shallow subtidal zone of all beaches (28–36 species) except for Venados Island (55 species), which had the highest number of species restricted to this island (7) (Table 3).

**Table 2.** Systematic list of species and sampling method used in the different environments of Bahía de Mazatlán, México. Q = quadrat & transect, D = dredge, DS = direct search, I = infaunal, E = epifaunal, S = semi-infaunal, En = endolithic; * = geographical range extensions; ** = species in only one environment; + = new record for the bay.

Species	Environments			Life forms
	Upper intertidal	Lower intertidal	Shallow subtidal	
**Mytilidae**				
1. *Brachidontes adamsianus* (Dunker, 1857)	Q	Q	Q, DS	E
2. *Brachidontes semilaevis* (Menke, 1849)	Q	Q	Q	E
3. *Gregariella coarctata* (P. P. Carpenter, 1857)	Q	Q	Q, D, DS	En
4. *Lioberus salvadoricus* (Hertlein & Strong, 1946)	-	-	Q, DS	E
5. *Lithophaga (Diberus) plumula* (Hanley, 1843) +	Q	Q	Q, D, DS	En
6. *Lithophaga (Labis) attenuata* (Deshayes, 1836)	Q	Q	Q, DS	En
7. *Lithophaga (Myoforceps) aristata* (Dillwyn, 1817)	Q	Q	Q, D, DS	En
8. *Lithophaga (Rupiphaga) hastasia* Olsson, 1961 *, +	-	-	Q, DS	En
9. *Adula soleniformis* (Olsson, 1961) *, +	-	-	Q, D, DS	En
10. *Botula cylista* S. S. Berry, 1959	-	Q	Q, D, DS	En
11. *Leiosolenus spatiosus* P. P. Carpenter, 1857	-	Q	Q, D, DS	En
12. *Modiolus americanus* (Leach, 1815)	-	-	Q, D	E
13. *Modiolus capax* Conrad, 1837	-	Q	Q	E
14. *Septifer zeteki* Hertlein & Strong, 1946 +	-	-	Q	E
**Arcidae**				
15. *Arca mutabilis* (G. B. Sowerby I, 1833)	Q	Q	D	E
16. *Arca pacifica* (G. B. Sowerby I, 1833) +	-	-	Q	E
17. *Acar bailyi* Bartsch, 1931	Q	-	-	E
18. *Acar gradata* Broderip & G. B. Sowerby I, 1829	Q	Q	Q, D, DS	E
19. *Acar rostae* (S. S. Berry, 1954)	Q	Q	Q, D, DS	E
20. *Barbatia reeveana* (d’Orbigny, 1846)	-	Q	-	E
21. *Barbatia illota* (G. B. Sowerby I, 1833) +	-	-	D	E
22. *Anadara formosa* (G. B. Sowerby I, 1833)	-	-	D, DS	S
**Noetiidae**				
23. *Arcopsis solida* (G. B. Sowerby I, 1833)	Q	Q	Q, D, DS	E
**Pteriidae**				
24. *Pinctada mazatlanica* (Hanley, 1856)	Q	Q	Q, DS	E
**Isognomonidae**				
25. *Isognomon (Melina) janus* P. P. Carpenter, 1857	Q	Q	Q, D, DS	E
26. *Isognomon (Melina) recognitus* (Mabille, 1895) +	-	Q	-	E
**Ostreidae**				
27. *Ostrea conchaphila* P. P. Carpenter, 1857	Q	Q	Q, DS	E
28. *Saccostrea palmula* (P. P. Carpenter, 1857)	Q	Q	Q, D, DS	E
29. *Striostrea prismatica* (J. E. Gray, 1825)	Q	Q	Q, D, DS	E
**Plicatulidae**				
30. *Plicatula penicillata* P. P. Carpenter, 1857	Q	-	Q, D, DS	E
31. *Plicatulostrea anomioides* (Keen, 1958)	Q	Q	Q, DS	E
**Limidae**				
32. *Limaria pacifica* (d’Orbigny, 1846)	-	Q	Q, DS	E
**Lucinidae**				
33. *Liralucina approximata* (Dall, 1901)	-	Q	-	S
34. *Ctena mexicana* (Dall, 1901) +	Q	Q	Q, D	S
**Carditidae**				
35. *Carditamera affinis* (G. B. Sowerby I, 1833)	Q	Q	Q, D, DS	I
36. *Cardites laticostatus* (G. B. Sowerby I, 1833)	Q	Q	Q, DS	I
**Crassatellidae**				
37. *Crassinella coxa* Olsson, 1964 +	-	-	Q	S
38. *Crassinella ecuadoriana* Olsson, 1961	-	Q	Q	S
39. *Crassinella nuculiformis* S. S. Berry, 1940 +	-	Q	Q	S
40. *Crassinella* aff. *pacifica* (C. B. Adams, 1852) +	Q	Q	Q	S
**Cardiidae**				
41. *Laevicardium substriatum* (Conrad, 1837) +	Q	-	-	I
**Chamidae**				
42. *Chama buddiana* C. B. Adams, 1852	Q	Q	Q, D, DS	E
43. *Chama coralloides* Reeve, 1846 +	Q	Q	Q, D, DS	E
44. *Chama sordida* Broderip, 1835	Q	Q	Q, DS	E
45. *Chama* cf. *frondosa* Broderip, 1835	-	-	D	E
**Lasaeidae**				
46. *Kellia suborbicularis* (Montagu, 1803) +	-	Q	Q, D, DS	En
**Mactridae**				
47. *Mactrellona subalata* (Mörch, 1860) *, +	-	-	D	I
48. *Mulinia pallida* (Broderip & G. B. Sowerby I, 1829) +	-	-	D	I
**Tellinidae**				
49. *Strigilla (Strigilla) cicercula* (R. A. Philippi, 1846)	-	Q	Q, D, DS	I
50. *Strigilla (Strigilla) dichotoma* (R. A. Philippi, 1846)	Q	Q	Q	I
51. *Strigilla (Strigilla) ervilia* (R. A. Philippi, 1846) *, +	Q	-	-	I
52. *Tellina (Laciolina) ochracea* P. P. Carpenter, 1864 +	-	-	Q	I
53. *Tellina (Moerella) coani* Keen, 1971 +	-	Q	Q, D	I
54. *Tellina (Moerella) felix* Hanley, 1844	-	Q	D	I
**Donacidae**				
55. *Donax (Chion) punctatostriatus* Hanley, 1843 +	Q	Q	-	I
56. *Donax (Paradonax) gracilis* Hanley, 1845	-	Q	D	I
**Semelidae**				
57. *Cumingia lamellosa* G. B. Sowerby I, 1833	Q	Q	-	I
58. *Semele (Semele)* cf. *bicolor* (C. B. Adams, 1852)	Q	-	-	I
59. *Semele (Semele) californica* (Reeve, 1853) +	-	-	Q	I
60. *Semele (Semele) flavescens* (A. A. Gould 1851) +	-	Q	-	I
61. *Semele jovis* (Reeve 1853) +	Q	-	-	I
62. *Semele hanleyi* Angas, 1879 +	Q	Q	Q	I
**Ungulinidae**				
63. *Diplodonta orbella* (A. A. Gould, 1851) +	Q	-	Q, D, DS	En
64. *Diplodonta (Pegmapex) caelata* (Reeve, 1850)	-	Q	Q, D, DS	En
65. *Diplodonta (Timothynus) inezensis* (Hertlein & Strong, 1947) +	-	-	Q	En
**Veneridae**				
66. *Chione subimbricata* (G. B. Sowerby I, 1835)	Q	Q	Q, D	S
67. *Chione undatella* (G. B. Sowerby I, 1835) +	-	-	Q	S
68. *Chioneryx squamosa* (P. P. Carpenter, 1857) +	Q	Q	Q	S
69. *Paphonotia elliptica* (G. B. Sowerby, 1834)	Q	-	-	S
70. *Periglypta multicostata* (G. B. Sowerby, 1835) +	Q	-	-	S
71. *Megapitaria squalida* (G. B. Sowerby, 1835)	-	-	Q	I
72. *Nutricola* cf. *humilis* (P. P. Carpenter, 1857)	-	-	D	S
73. *Pitar* cf. *omissa* (Pilsbry & Lowe, 1932)	-	-	Q	I
74. *Transennella modesta* (G. B. Sowerby, 1835) +	-	-	Q	S
75. *Transennella* cf. *puella* (P. P. Carpenter, 1864)	-	-	Q, D	S
**Neoleptonidae**				
76. *Neolepton (Neolepton) subtrigonum* (P. P. Carpenter, 1857)	-	Q	Q, D, DS	S
**Myidae**				
77. *Sphenia fragilis* (H. & A. Adams 1854) +	Q	Q	Q, D, DS	En
**Corbulidae**				
78. *Caryocorbula biradiata* (G. B. Sowerby I, 1833)	Q	Q	Q	I
79. *Caryocorbula marmorata* (Hinds, 1843) +	Q	Q	Q, DS	I
80. *Caryocorbula nasuta* G. B. Sowerby I, 1833	Q	-	Q, D	I
81. *Juliacorbula bicarinata* G. B. Sowerby I, 1833	Q	Q	Q	I
**Petricolidae**				
82. *Choristodon robustus* (G. B. Sowerby I, 1834) +	-	-	D	En
83. *Petricola (Petricola) linguafelis* (P. P. Carpenter, 1857) +	-	Q	Q, D, DS	En
84. *Petricola (Petricolirus) californiensis* Pilsbry & Lowe, 1932 +	-	-	DS	En
**Phadidae**				
85. *Parapholas calva* (G. B. Sowerby I, 1834) +	-	-	Q	En
86. *Pholadidea (Hatasia) melanura* (G. B. Sowerby I, 1834)	-	-	D	En
**Hiatellidae**				
87. *Hiatella arctica* (Linnaeus, 1767)	Q	Q	Q, D, DS	En
**Gastrochaenidae**				
88. *Lamychaena truncata* (G. B. Sowerby I, 1834)	-	-	Q, D	En
**Lyonsidae**				
89. *Entodesma brevifrons* (G. B. Sowerby I, 1834) +	-	Q	Q, D, DS	I
**Total sampling species richness** (**)	**44 (7)**	**53 (4)**	**76** **Q = 64 (10)** **D = 42 (7)** **DS = 38 (1)**	-
**Total infaunal species richness**	-			**26**
**Total semi-infaunal species richness**	-			**16**
**Total endolithic species richness**	-			**20**
**Total epifaunal species richness**	-			**27**

**Table 3.** Distribution of bivalve species at six sites in the Bahía de Mazatlán, México. Sites: OA = Olas Altas, LP = Los Pinos, CM = Casa del Marino, VI = Venados Island, MA = Malecon, ZD = Zona Dorada. Environments: UI = upper intertidal, LI = lower intertidal, SS = shallow subtidal, * = species recorded in only one site or environment. AF = absolute frequency, by sites and environment (for each environment, the number of sites where there was a species / total sites).

Species	Sites and Environments																				
	OA			LP			CM			VI			ZD			MA			AF		
	UI	LI	SS	UI	LI	SS	UI	LI	SS	UI	LI	SS	UI	LI	SS	UI	LI	SS	UI	LI	SS
*Acar bailyi*	X			X			X												0.5	-	-
*Acar gradata*	X	X		X	X	X	X	X	X		X	X			X				0.5	0.7	0.7
*Acar rostae*	X	X	X	X	X	X	X	X	X	X	X	X			X			X	0.7	0.7	1.0
*Adula soleniformis*															X			X	-	-	0.3
*Anadara formosa*												X						X	-	-	0.3
*Arca mutabilis*				X	X		X	X			X							X	0.3	0.5	0.2
Arca pacifica*						X													-	-	0.2
*Arcopsis solida*	X	X	X	X	X	X	X	X	X	X	X	X							0.7	0.7	0.7
Barbatia illota*																		X	-	-	0.2
Barbatia reeveana*								X											-	0.2	-
*Botula cylista*		X				X			X			X			X			X	-	0.2	0.8
*Brachidontes adamsianus*	X	X		X	X	X	X	X	X	X	X	X			X				0.7	0.7	0.7
*Brachidontes semilaevis*	X	X	X	X	X	X	X	X		X	X	X							0.7	0.7	0.5
*Carditamera affinis*	X	X	X	X	X	X	X	X	X	X	X	X			X			X	0.7	0.7	1.0
*Cardites laticostatus*	X	X			X	X				X	X	X						X	0.3	0.5	0.5
*Caryocorbula biradiata*	X	X		X	X		X	X	X										0.5	0.5	0.2
*Caryocorbula marmorata*						X	X	X	X		X	X							0.2	0.3	0.5
*Caryocorbula nasuta*			X				X					X						X	0.2	-	0.5
*Chama buddiana*		X	X	X	X	X	X	X	X		X	X						X	0.3	0.7	0.8
*Chama* cf. frondosa*												X							-	-	0.2
*Chama coralloides*	X	X	X	X	X	X			X	X	X	X			X				0.5	0.5	0.8
*Chama sordida*	X	X		X		X	X		X		X	X							0.5	0.3	0.5
*Chione subimbricata*	X	X		X		X			X	X	X	X							0.5	0.3	0.5
Chione undatella*												X							-	-	0.2
*Chioneryx squamosa*	X					X					X	X							0.2	0.2	0.3
Choristodon robustus*															X				-	-	0.2
*Crassinella* aff. *pacifica*										X	X	X							0.2	0.2	0.2
Crassinella coxa*									X										-	-	0.2
*Crassinella ecuadoriana*									X		X	X			X				-	0.2	0.5
*Crassinella nuculiformis*					X							X							-	0.2	0.2
*Ctena mexicana*							X	X				X			X			X	0.2	0.2	0.5
*Cumingia lamellosa*				X	X														0.2	0.2	-
*Diplodonta caelata*					X	X		X	X			X			X			X	-	0.3	0.8
*Diplodonta inezensis*									X			X							-	-	0.3
*Diplodonta orbella*	X			X					X			X						X	0.3	-	0.5
*Donax gracilis*		X																X	-	0.2	0.2
*Donax punctatostriatus*													X	X		X	X		0.3	0.3	-
*Entodesma brevifrons*											X	X			X			X	-	0.2	0.5
*Gregariella coarctata*	X	X				X			X		X	X			X			X	0.2	0.3	0.8
*Hiatella arctica*	X	X	X		X	X			X		X	X			X			X	0.2	0.5	1.0
*Isognomon janus*	X	X	X	X	X	X	X	X	X	X	X	X						X	0.7	0.7	0.8
Isognomon recognitus*								X											-	0.2	-
*Juliacorbula bicarinata*	X	X		X		X	X	X											0.5	0.3	0.2
*Kellia suborbicularis*			X		X	X			X			X			X				-	0.2	0.8
Laevicardium substriatum*	X																		0.2	-	-
*Lamychaena truncata*			X						X			X						X	-	-	0.7
*Leiosolenus spatiosus*		X	X		X	X			X		X	X			X			X	-	0.5	1.0
*Limaria pacifica*						X		X				X							-	0.2	0.3
*Lioberus salvadoricus*						X						X							-	-	0.3
Liralucina approximata*											X								-	0.2	-
*Lithophaga aristata*	X	X	X	X	X	X			X	X	X	X			X			X	0.5	0.5	1.0
*Lithophaga attenuata*	X	X	X	X	X	X	X		X			X			X				0.5	0.3	0.8
Lithophaga hastasia*															X				-	-	0.2
*Lithophaga plumula*		X	X			X			X	X	X	X			X			X	0.2	0.3	1.0
*Mactrellona subalata*			X												X				-	-	0.3
Megapitaria squalida*												X							-	-	0.2
*Modiolus americanus*									X			X							-	-	0.3
*Modiolus capax*			X		X				X		X	X							-	0.3	0.5
Mulinia pallida*			X																-	-	0.2
*Neolepton subtrigonum*			X		X	X			X		X	X			X				-	0.3	0.8
*Nutricola* cf. humilis*			X																-	-	0.2
*Ostrea conchaphila*	X	X	X	X	X	X	X	X		X	X	X			X			X	0.7	0.7	0.8
Paphonotia elliptica*	X																		0.2	-	-
Parapholas calva*												X							-	-	0.2
Periglypta multicostata*				X															0.2	-	-
Petricola californiensis*																		X	-	-	0.2
*Petricola linguafelis*					X	X			X		X	X			X			X	-	0.3	0.8
Pholadidea melanura*																		X	-	-	0.2
*Pinctada mazatlanica*	X				X				X			X							0.2	0.2	0.3
*Pitar* cf. omissa*			X																-	-	0.2
*Plicatula penicillata*	X														X			X	0.2	-	0.3
*Plicatulostrea anomioides*	X	X		X	X	X	X	X	X	X	X	X			X				0.7	0.7	0.7
*Saccostrea palmula*	X	X	X	X	X	X	X	X	X	X	X	X			X			X	0.7	0.7	1.0
*Semele californica*			X						X										-	-	0.3
*Semele* cf. bicolor*							X												0.2	-	-
*Semele flavescens*		X			X			X											-	0.5	-
*Semele hanleyi*										X	X	X							0.2	0.2	0.2
Semele jovis*				X															0.2	-	-
Septifer zeteki*												X							-	-	0.2
*Sphenia fragilis*	X	X	X		X				X		X	X			X			X	0.2	0.5	0.8
*Strigilla cicercula*			X											X	X			X	-	0.2	0.5
*Strigilla dichotoma*			X										X	X					0.2	0.2	0.2
Strigilla ervilia*	X																		0.2	-	-
*Striostrea prismatica*	X	X	X	X	X	X	X	X	X	X	X	X			X			X	0.7	0.7	1.0
*Tellina coani*					X	X												X	-	0.2	0.3
*Tellina felix*					X													X	-	0.2	0.2
Tellina ochracea*												X							-	-	0.2
*Transennella* cf. puella*			X																-	-	0.2
Transennella modesta*												X							-	-	0.2
**Exclusive species**	**3**	-	**4**	**2**	-	**1**	**1**	**2**	**1**	-	**1**	**7**	-	-	**2**	-	-	**2**			
**Total (enviroment by site)**	**30**	**28**	**28**	**25**	**32**	**34**	**22**	**22**	**36**	**17**	**33**	**53**	**2**	**3**	**30**	**1**	**1**	**33**			
**Total epifaunal species richness**	**17**	**15**	**10**	**16**	**17**	**18**	**15**	**16**	**15**	**12**	**17**	**22**	-	-	**10**	-	-	**11**			
**Total endolithic species richness**	**6**	**8**	**7**	**3**	**8**	**10**	**1**	**1**	**13**	**2**	**7**	**14**	-	-	**14**	-	-	**13**			
**Total semi-infaunal species richness**	**3**	**1**	**3**	**2**	**2**	**3**	**1**	**1**	**4**	**2**	**6**	**10**	-	-	**3**	-	-	**2**			
**Total infaunal species richness**	**4**	**4**	**8**	**4**	**5**	**3**	**5**	**4**	**4**	**1**	**3**	**7**	**2**	**3**	**3**	**1**	**1**	**7**			
**Total species richness by sites**	**48**			**47**			**49**			**55**			**32**			**34**					

Several species of small size (5–10 mm) were recorded in rocky and sandy substrates: *Crassinella coxa*, *Crassinella nuculiformis*, *Liralucina approximata*, *Ctena mexicana*, *Kellia suborbicularis*, *Neolepton subtrigonum*, *Nutricola* cf. *humilis*, *Pitar* cf. *omissa*, *Sphenia fragilis*, *Chioneryx squamosa*, and *Transennella* cf. *puella*. Only three species were collected in the sandy intertidal environment: *Strigilla cicercula*, *Strigilla dichotoma*, and *Donax punctatostriatus*.

More species were recorded with the quadrat-transect technique in the shallow subtidal (64 species) than with either dredges (42 species) or direct searches (38 species). However, the species composition was different since some were collected only with quadrat-transects (10 species), dredges (7) or direct searches (1). The life-forms recorded included epifaunal (27), infaunal (26 species), endolithic (20), and semi-infaunal (16). Endolithic species were found in various hard substrates such as sedimentary rocks, corals, polychaete tubes, bivalve shells, and rodoliths. The rock-drilling bivalve *Parapholas calva* was found only in sedimentary rocks; all other species were found in two or more types of hard substrates.

Compared to previous studies in the region, this study includes 37 new records for Bahía de Mazatlán (Table 1), and geographic range extensions for four species: *Lithophaga hastasia*, *Adula soleniformis*, *Mactrellona subalata*, and *Strigilla ervilia*.

Twelve (13.5%) of the 89 species recorded were widely distributed in the bay (e.g., found in six sites), and 11 were recorded at three of the environments (UI, LI, and SS): *Acar rostae*, *Carditamera affinis*, *Gregariella coarctata*, *Hiatella arctica*, *Lithophaga aristata*, *Lithophaga plumula*, *Leiosolenus spatiosus*, *Ostrea conchaphila*, *Saccostrea palmula*, *Striostrea prismatica*, and *Sphenia fragilis*. However, a large number of species (27) were unique to one environment and sampling site. Only nine species were recorded in the three environments and in five or six sampling sites: *Acar rostae*, *Arcopsis solida*, *Brachidontes adamsianus*, *Carditamera affinis*, *Isognomon janus*, *Ostrea conchaphila*, *Plicatulostrea anomioides*, *Saccostrea palmula*, and *Striostrea prismatica* (Table 3).

There are several distribution patterns revealed by the life forms recorded in the different environments of Bahía de Mazatlán. Epifaunal bivalves were more frequent in the upper and lower rocky intertidal (12–17 species), followed by the endolithic species (1–8), whereas the number of infaunal species was very similar in all six sites (4–5), except for Venados Island. Similarly, epifaunal species dominated the shallow subtidal and intertidal zones (10–22), followed by the endolithic (7–14), infaunal (3–8) and semi-infaunal species (2–10) (Table 3).

### Taxonomic distinctness

**Figure 4.**
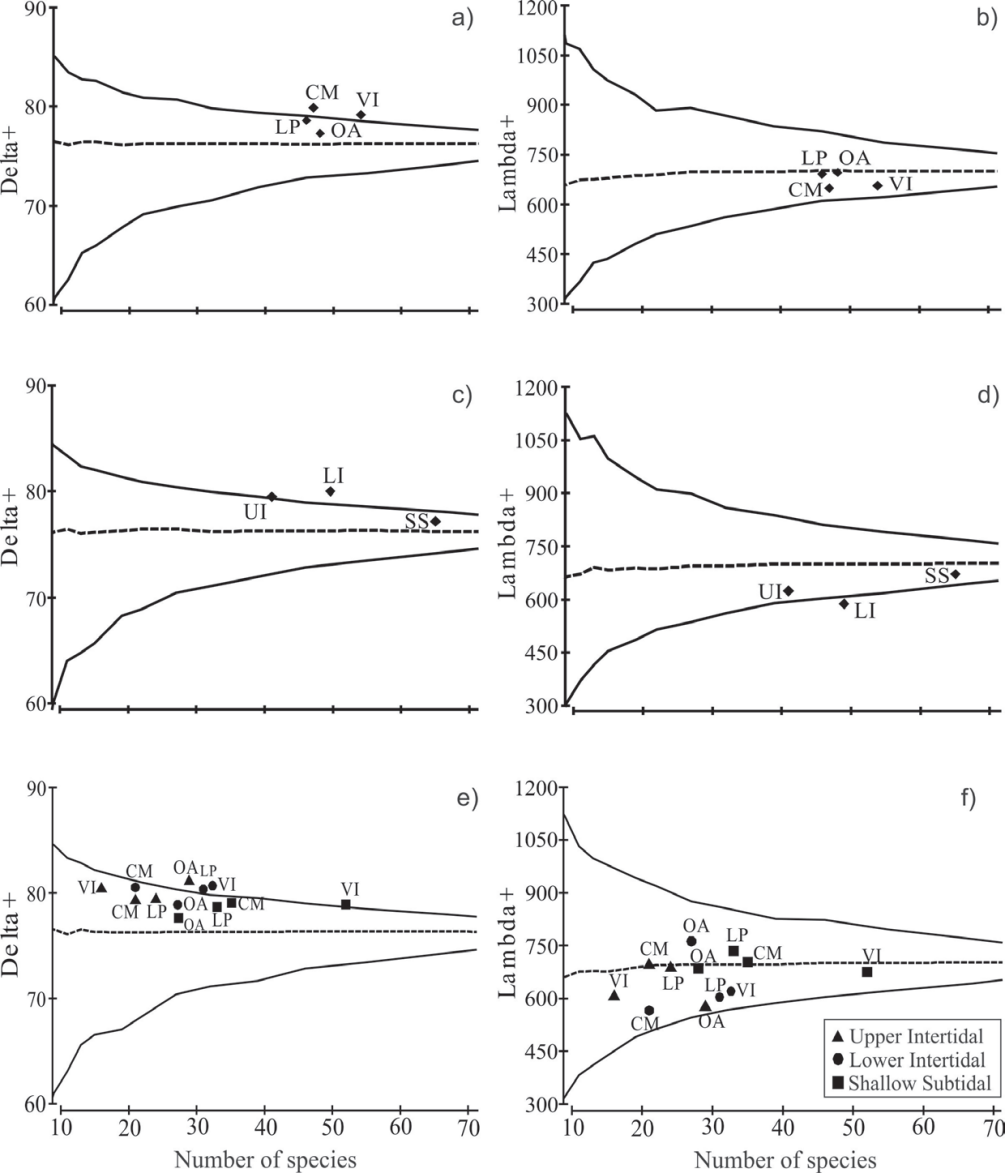
Average taxonomic distinctness (∆^+^) and variation in taxonomic distinctness (Λ^+^) of bivalves assemblages in the four sites (**a** and **b**), in the three environments (**c** and **d**) and in the sites by environments of Bahía de Mazatlán (**e** and **f**). The continuous line shows confidence intervals at 95% and the dashed line shows values ∆^+^ & Λ^+^. The statistical significance of ∆^+^ & Λ^+^ were tested using 1,000 permutations. Abbreviations as in Table 3.

The average taxonomic distinctness analysis revealed complementary information on the bivalve assemblages recorded in the sampling sites and in the environments. The values of Δ^+^ for Los Pinos and Olas Altas fell within the probability funnel (e.g. within the confidence intervals of 95%, p>0.05), indicating a greater contribution to the mean taxonomic diversity of Bahía Mazatlan. However, values of Λ^+^ fell within the probability funnel for the four sites, suggesting that these are significantly representative of the bay’s bivalve assemblage (Figures 4a–b). On the other hand, the values of Δ^+^ for the SS zone and the values of Λ^+^ of the UI and SS fell within the confidence funnel, close to the bay’s mean taxonomic inventory (Figures 4c–d). Finally, in the case of the site-by-environment analysis, most sites fell within the Δ^+^ probability funnel (p>0.05), except for Olas Altas upper intertidal zone and Los Pinos and Venados Island lower intertidal zone (Figure 4e). Also the Λ^+^ values of all sites and environments fell within the funnel, i.e., all sites contribute significantly to the bay’s total taxonomic diversity (Figure 4f; Table 4).

**Table 4.** Number of bivalve species, genera, families, superfamilies, orders and superorders registered in four sites in Bahía de Mazatlán, México. Sites: OA = Olas Altas, LP = Los Pinos, CM = Casa del Marino, VI = Venados Island. Environments: UI = upper intertidal, LI = lower intertidal, SS = shallow subtidal.

Classification levels	Taxon	OA			LP			CM			VI		
		UI	LI	SS	UI	LI	SS	UI	LI	SS	UI	LI	SS
1	Species	30	28	28	25	32	34	22	22	36	17	33	53
2	Genera	25	22	24	19	26	28	16	18	26	15	27	39
3	Families	14	14	17	13	19	14	11	13	19	11	18	24
4	Superfamilies	11	12	13	11	15	14	10	12	15	10	14	19
5	Orders	9	9	9	8	9	10	9	10	10	7	10	12
6	Superorders	2	2	2	2	2	2	2	2	2	2	2	2

## Discussion

The present work increased significantly (31%) the inventory of bivalve species of Bahía de Mazatlán to an updated total number of 132 species, including 37 new records (Table 5). According to these figures, the bay contributes 34% to the bivalve diversity of the southern Golfo de California (390 species) (Hendrickx et al. 2007), and approximately 15% to the Eastern Tropical Pacific region (890 species) (Coan and Valentich-Scott 2012). The intensive sampling strategy applied during this survey contributed considerably to the thoroughness of the inventory. In the subtidal zone, the Van Veen dredge and trawl net that had been used in previous studies failed to collect many epifaunal and endolithic life forms that we obtained during SCUBA diving in all six sites of the bay. Also, the number of species previously recorded in the intertidal zone (9–19) by using a single sampling technique, either quadrats, quadrat-transects, transects, or direct searches (Arreguín-Romero 1982, Sanchez-Vargas 1984, Camacho-Montoya et al. 2007, Vega et al. 2008, Rendón-Díaz 2010), was increased to 63 by using a combination of techniques during the four sampling expeditions throughout the year. The distribution patterns of bivalves in the two main environments of the bay, the intertidal and shallow intertidal, are also extended with the records of eight species previously known only from the subtidal zone and recorded for the first time in the intertidal zone of the bay: *Caryocorbula biradiata*, *Caryocorbula nasuta*, *Donax gracilis*, *Gregariella coarctata*, *Strigilla cicercula*, *Strigilla dichotoma*, and *Parvilucina aproximata* (Orozco-Romo 1980, Sánchez-Vargas 1984); and the records of 16 bivalve species in the shallow subtidal, previously reported only in the intertidal zone (Arreguín-Romero 1982, Sánchez-Vargas 1984, Olabarría et al. 2001, Camacho-Montoya et al. 2007, Rendón-Díaz 2010).

**Table 5.** Previous studies in Bahía de Mazatlán, México. * = Species list not provided.

Environments	Sampling method	Total species	Shared species	Reference
Subtidal (10–15 m)	Grab (Van Veen)	3	2	Parker (1963)
Subtidal (3.5–27 m)	Grab (Van Veen)	42	11	Orozco-Romo (1980)
Subtidal	Trawls	2	1	
Rocky intertidal	Quadrats & transects	15	14	Arreguín-Romero (1982)
Middle rocky intertidal	Direct search	4	4	Sánchez-Vargas (1984)
Lower rocky intertidal	Direct search	12	11	
Shallow subtidal (1–5 m)	Grab (Van Veen) & trawls	22	12	
Rocky– sandy intertidal	Quadrats	7	5	Olabarria et al. (2001)
Rocky– sandy intertidal	Quadrats	13	5	Camacho-Montoya et al. (2007)
Intertidal	Quadrats & transects	9	-	Vega et al. (2008)*
Rocky– sandy intertidal	Transect band	19	13	Rendón-Díaz (2010)
**Total species previous studies**		**83**		
**Total shared species**			**40**	
**Rocky– sandy intertidal**	**Quadrats & transects**	**60**		**This study**
**Sandy intertidal**	**Quadrats & transects**	**3**		
**Subtidal (4–10 m)**	**Quadrats & transects**	**64**		
**Subtidal (8–15 m)**	**Naturalist’s dredge**	**42**		
**Subtidal (4–10 m)**	**Direct search**	**38**		
**Total species present study**		**89**		
**Total species in Bahía de Mazatlán**		**132**		

Our surveys yielded a substantial increase in the number of infaunal (29%) and endolithic (23%) species of bivalves; most of them (67%) not recorded previously in the bay. This is particularly important since frequently the species richness of molluscs has been underestimated in ecological investigations due to two main factors that, alone or combined, contribute to incomplete inventories (Bouchet et al. 2002). The first factor is inadequate coverage of the spatial heterogeneity, due to inappropriate sampling techniques. These limitations result in missing specialized species that live in a limited or specific area or habitat. The second factor is the overvaluation of macromolluscs (i.e., collection only of conspicuous species): studies that include species inventories tend to focus on large species (≥ 10 mm) and ignore the small ones. One reason for excluding molluscs less than 10 mm in size is the difficulty for taxonomic determination. Another factor that contributes to the exclusion of small molluscs from ecological studies is the failure of a detailed review of the sediments where bivalves in this size range are common. Therefore, it was important to address these factors for a more complete inventory of bivalves.

We also report range extension of four species previously known in other regions of the Eastern Pacific coast: *Lithophaga hastasia* (from Bahía de Banderas, Jalisco, to Perú); *Strigilla ervilia* (from Bahía de Tenacatita, Jalisco, to Salinas, Ecuador); *Mactrellona subalata* (from La Peñita, Nayarit to Tumbes, Perú); and *Adula soleniformis* (El Lagartillo, Los Santos, Panamá to Paita, Perú) (Coan and Valentich-Scott 2012).

A total of 83 additional species were collected during field work; these are not reported here because they were not living specimens however they were identified from complete and well preserved shells. Interestingly, most of these species (64) have not been recorded previously in the bay, thus raising the total inventory (living specimens plus empty shells) to 196 species. Many ecological investigations include the species recorded from empty mollusc shells assuming that they are components of the regional community (i.e., Warwick and Light 2002, Smith 2008). However, at the local scale (i.e., sites, environments), most authors exclude them, arguing that empty shells may be transported by both currents and invertebrates (i.e. hermit crabs) so their presence may be incidental and there is no guarantee that these empty shells are part of the community at the time of collecting (Bouchet et al. 2002). Thus, on a regional scale (i.e., Bahía del Mazatlán) this complete inventory (196 species) including empty shells and live specimens may be taken into consideration. However, since the composition of the assemblage is described here in a more detailed way and it was associated with sampling sites and specific environments, we decided to exclude the empty shells and define the assemblage of bivalves in a conservative way using only live specimen associated with a narrow vertical distribution range which includes two adjacent interconnected environments of the bay: the intertidal and shallow subtidal (< 10 m depth).

**Figure 5.**
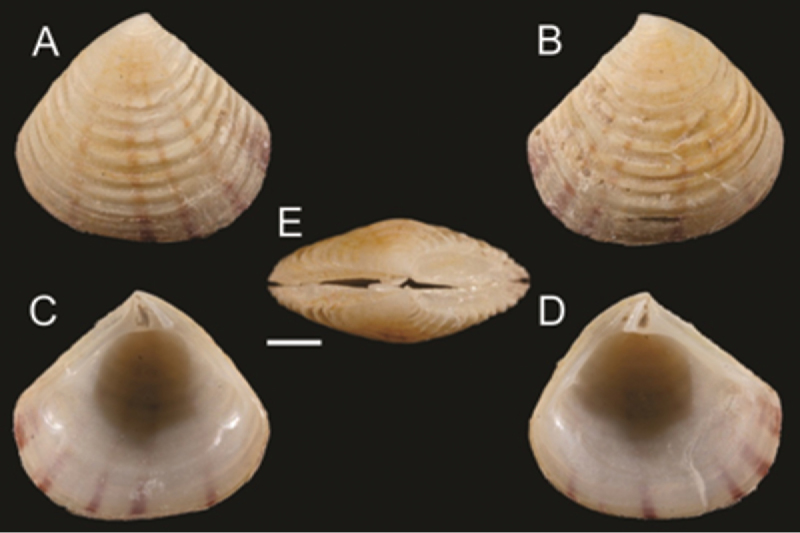
*Crassinella* aff. *pacifica*. Length = 4.92 mm **A** Exterior of right valve **B** Exterior of left valve **C** Interior of right valve **D** Interior of left valve **E** Dorsal view of both valves joined. Scale = 1 mm. Venados Island, Bahía de Mazatlán, México. LEMA-BI-14. Photography credit: Paul Valentich-Scott.

Some implications that emerge from the taxonomic identification of five bivalve taxa classified here as “cf.” (from the Latin *confer* which means “compare with”, that is, similar to and probably the same as, the parent taxon) are worth mentioning. These specimens corresponded to juvenile stages (*Chama* cf. *frondosa*, *Semele* cf. *bicolor*, *Nutricola* cf. *humilis*, *Transennella* cf. *puella*, and *Pitar* cf. *omissa*), which restricts their taxonomic determination because the keys and photographs in the literature consistently refer to adult specimens (i.e., Keen 1971, Coan and Valentich-Scott 2012). However, the juvenile specimens collected displayed certain distinctive features that prove their resemblance to that species. The specimens of *Crassinella* aff. *pacifica*, collected in Venados Island, have all the characteristics of the species, although some of them display a slight variation in the beak that does not correspond to the taxon. Therefore, for practical purposes all individuals collected in this study in all sites were determined as *Crassinella* aff. *pacifica*. Nonetheless, these specimens warrant a detailed examination to rule out a potential new species (Figure 5A–E).

Some specimens of the rock oyster *Striostrea prismatica* did not show the thick lamellae on the outer shell surface which characterize this species. Instead, they exhibited tubular spines as *Ostrea tubulifera*. The spines are located on the outer edge of the shell, and the features of the inner surface match those of *Striostrea prismatica*. If only the analysis of morphological traits is considered, the problem could be explained as either hybridization between the two species–as this phenomenon is very common among oysters (Leitão et al. 2007), or an atypical species trait, or ontogenetic variation. Only a genetic analysis could resolve the true identity.

According to the different projections obtained with species accumulation curves, the expected total numbers of species is 32% (for the intertidal zone) and 57% (for the subtidal zone) higher than the number of species we actually collected. This difference relates to the large number of rare species recorded and it is a good estimator of the potential number of species expected in these environments at Bahía de Mazatlán. Even so, the species accumulation curves confirmed that our sampling effort was sufficient to calculate the theoretical total number of bivalve species in the bay.

Although different sampling techniques were used in the bay’s different environments, the sampling effort was estimated only for the quadrat-transect technique. Thus, whether all the bivalve species that inhabit the bay were collected in this study was not satisfactorily demonstrated. Some bivalves may be present either only in some seasons or impermanently, so these will not be recorded irrespective of the sampling intensity, which in turn is reflected in the sampling effort outcome (Figures 2, 3).

The high marine biodiversity of Golfo de California has been related to its irregular coastal geomorphology (i.e., open and protected bays and inlets, rocky and sandy beaches, estuaries, and numerous islands), the local dynamics of the surface currents and the seabed heterogeneity (Hendrickx and Brusca 2002, Hendrickx et al. 2005). According to Roy et al. (2000), there is a remarkable increase in infaunal and epifaunal bivalve species in the northeastern Pacific coast, between latitudes 5°N (i.e., Punta Paita, Perú) and 23°N (i.e., Bahía de Mazatlán, México). The bivalve species richness in Golfo de California has been documented by Parker (1963) who reported 380 species; Hendrickx et al. (2007) who reported 565 species; and Zamorano and Hendrickx (2007) who reported 137 species. Therefore, Bahía de Mazatlán has approximately 23% of the bivalve species reported for this region. Coan (1968) recorded 75 bivalve species in Bahía de los Ángeles, which has a similar size to Bahía de Mazatlán and it is located in the northern portion of the Golfo de California. Many of the bivalves from Bahía de los Ángeles are infaunal forms mostly associated with sandy-silt substrates which prevail in this bay. A detailed review of the malacological fauna of these bays indicates that they are quite different with only 12 bivalve species shared. According to Hendrickx et al. (2007), invertebrate diversity generally tends to decrease from south to north in the Golfo de California. Actually, these authors document a reduction in the number of bivalve species along a south-to-north latitudinal gradient of the gulf. The region is generally considered to be warm temperate with a combination of elements from two adjacent provinces and ecoregions: the Cortezian Ecoregion, in the southern end of the Warm Temperate Northeast Pacific province and the Mexican Tropical Pacific Ecoregion, in the northern part of the Tropical Eastern Pacific province (Spalding et al. 2007).

The characteristics of the Bahía de Mazatlán coastline provide a variety of benthic habitats to support a large number of bivalve species. A number of studies on the Mexican Pacific coast have shown that the high species richness and diversity of bivalve life forms are related to substrate heterogeneity, wave exposure and particle size of the sediments in the intertidal and shallow subtidal environments (Parker 1963, Coan 1968, Esqueda et al. 2000, González-Medina et al. 2006, Hendrickx et al. 2007, Ríos-Jara et al. 2008, Vega et al. 2008, López-Uriarte et al. 2009, Ríos-Jara et al. 2009). Bahía de Mazatlán comprises islands, rocky reefs, small aggregations of coral, rocky and sandy shores, all of which increase the heterogeneity and availability of marine habitats. In addition, there are two well-defined seasons throughout the annual cycle – the dry season and the wet season – with major changes in primary productivity, nutrients, and phytoplankton (Alonso-Rodríguez 2004). This environmental heterogeneity contributes to the presence of numerous bivalve species and life forms, and contrasts with the low richness observed by Parker (1963) in deeper bays (> 10 m) with more homogeneous soft bottoms (Orozco-Romo 1980).

Our analysis combined data from three different sampling techniques, which was a major advantage, as the average taxonomic distinctness analysis is not affected by the various techniques and sampling effort used (Warwick and Clarke 2001, Leonard et al. 2006). With this method we identified the sites and environments that, according to their species taxonomic composition, are within the 95% probability funnel of average taxonomic distinctness (∆^+^) and its variation (Λ^+^). Their inclusion within the probability funnel indicates that they involve a good representativeness of the bay’s taxonomic diversity.

In this study, the combination of sites and environments provided better values of Δ^+^ and Λ^+^ when rocky shores and shallow subtidal adjacent zones were taken into consideration. This is because, although the three environments are clearly different from each other, all the sites contribute towards the taxonomic diversity of the bay. For example, Venados Island and Casa del Marino had the highest number of taxa in the shallow subtidal zone; Los Pinos and Venados Island, in the lower intertidal zone; and Olas Altas, in the upper intertidal zone. Theoretically, populations with a high genetic diversity have a high evolutionary potential or ability to adapt to changing environmental conditions (Price 2002). Further comparative studies among regions, clades, and functional groups are needed to understand the bivalve assemblage of Bahía de Mazatlán.

## Conclusion

The present work demonstrates that the bivalve fauna in Bahía de Mazatlán is well represented by various life forms (epifaunal, infaunal, semi-infaunal, and endolithic) in all the sites studied. Venados Island is an area protected by two government agencies; this is significant because it displayed high species richness and a large number of unique species. Since the bay is now a popular destination for tourists, efforts to preserve its ecosystems and species are essential, including those bivalves of economic importance such as the rock oyster *Striostrea prismatica* and the pearl oyster *Pinctada mazatlanica*. The latter species is on the Mexican Official List of Protected Species (NOM-059-SEMARNAT-2010).

The information on bivalve assemblages in Bahía de Mazatlán should be supplemented with analysis including an assessment of α, γ, and β diversity in order to determine their relative distribution at different spatial scales. A quantitative analysis investigating the relationship between bivalve assemblage structure and local and seasonal environmental parameters is also required. Such an analysis, would contribute to a comprehensive framework on the ecology of these bivalves, which is essential for further studies on the conservation of the bay.
